# Significance of Capping Agents of Colloidal Nanoparticles from the Perspective of Drug and Gene Delivery, Bioimaging, and Biosensing: An Insight

**DOI:** 10.3390/ijms231810521

**Published:** 2022-09-10

**Authors:** Rabia Javed, Anila Sajjad, Sania Naz, Humna Sajjad, Qiang Ao

**Affiliations:** 1 NMPA Key Laboratory for Quality Research and Control of Tissue Regenerative Biomaterial, Institute of Regulatory Science for Medical Device, National Engineering Research Center for Biomaterials, Sichuan University, Chengdu 610065, China; 2 Department of Biotechnology, Quaid-i-Azam University, Islamabad 45320, Pakistan

**Keywords:** capping agents, nanoparticles, therapeutics, drug delivery, diagnostics, biosensing

## Abstract

The over-growth and coagulation of nanoparticles is prevented using capping agents by the production of stearic effect that plays a pivotal role in stabilizing the interface. This strategy of coating the nanoparticles’ surface with capping agents is an emerging trend in assembling multipurpose nanoparticles that is beneficial for improving their physicochemical and biological behavior. The enhancement of reactivity and negligible toxicity is the outcome. In this review article, an attempt has been made to introduce the significance of different capping agents in the preparation of nanoparticles. Most importantly, we have highlighted the recent progress, existing roadblocks, and upcoming opportunities of using surface modified nanoparticles in nanomedicine from the drug and gene delivery, bioimaging, and biosensing perspectives.

## 1. Introduction

The manipulation of matter such that it has size dimensions ranging from 1 to 100 nm is the scientific discipline termed as nanotechnology, and the particles having dimensions on the nanoscale are called nanoparticles. In the past few decades, nanotechnology is booming with immense success and doing innovations in the areas of medicine, electronics, energy, environment, and agriculture due to the distinctive physicochemical, optical, electrical, magnetic, and biological characteristics of the nanomaterials [1,2]. The nanoparticles that are aggregated colloidal solutions are unsafe and toxic for living cells. These nanoparticles, if coated with capping agents, alter their surface composition and size distribution. It makes the nanoparticles more compact, resulting in the mitigation of aggregation, and amelioration of biocompatibility and non-toxicity by stopping their non-specific interaction [3].

The capping agents used for modifying nanoparticles are selected on the basis of their prominent biological properties like biocompatibility, biodegradability, bioavailability, and biosolubility so that the applicability of such capped nanomaterials could be enhanced in the living cellular environment. The small-sized nanoparticles can easily float in the human body in comparison to their larger counterparts, therefore selected for nanomedical purposes [4,5]. The chains of capping moieties are attached to nanoparticles’ outer surface by means of covalent or non-covalent forces of attraction which provides them stability by either stearic hindrance (due to the presence of long organic/bulky polymeric chains) or electrostatic repulsion (due to the cationic or anionic nature of capping agents) [6]. The agglomeration between nanoparticles is prevented making them more stable by the effect of capping. The surface activation of nanoparticles makes them available for binding to other molecules. Their shelf-life increases and are protected against moisture absorption or other such chemical reactions. Moreover, the rising concerns about nanosafety are also lessened to some extent by adopting this strategy [7].

The amphiphilic structure of capping agents involves a polar head attached to nanoparticles’ surfaces and a non-polar tail immersed in surrounding medium. The capping agents further increase the total percentile of atoms found on the surface of nanoparticles, conferring them escalation in reactivity such as biological functioning [8]. The tailoring of colloidal nanoparticles’ suspensions with respect to their size, morphology, and surface composition occurs using appropriate capping agents that enables them to be used in a plethora of biomedical phenomenon such as bioimaging and biosensing. These are the areas that have been substantially improved by employing capping agents in nanomaterials’ synthesis. Moreover, the reduced sized and surface modified nanoparticles have proven significantly important in targeted drug or gene delivery and immunotherapy. In this way, applications of capped nanomaterials in different domains of nanomedicine concerning diagnosis and therapeutics of devastating ailments like cancer, tuberculosis (TB), diabetes, cardiovascular diseases (CVD), and neurological defects are continuously being exploited [9].

This review provides an overview of the involvement of different capping agents in the fabrication of nanoparticles. Next, the potential impact of the capping agents for targeted drug and gene delivery, bioimaging, and biosensing has been elaborated with examples from recent literature. This study documenting the influence of capping agents in the functioning of colloidal nanoparticles in the context of different biomedical domains is much needed because it enables readers to understand why controlled size and definite shape/morphology is essential to obtain specified outcomes in the field of nanomedicine. It unfolds the necessity for stability, ameliorated reactivity, and escalated binding ability of capped nanoparticles to biological receptors, consequently improving the processes of bioimaging, biosensing, drug delivery, and gene delivery.

## 2. Preparation and Biomedical Evaluation of Capped Nanoparticles

**Figure 1:**
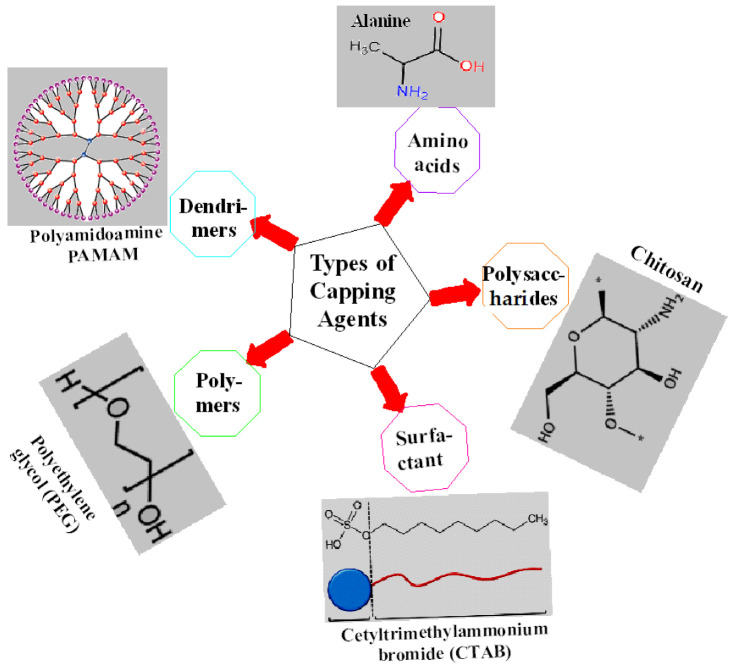
Different types of capping agents along with examples.

Capping agents can be broadly categorized into organic ligands, surfactants, polysaccharides, proteins, amino acids, polymers, dendrimers, cyclodextrins, bio-extracts, etc., few of which are shown by Figure 1. It is the intrinsic property of capping agents to become firmly adsorbed on the nanoparticles’ surface forming mono- or multi-layered protective coating that regulates their size, shape, and geometry, hence providing them long-term stability. All of these parameters govern the behavior of nanoparticles in biological systems; most importantly, the significant enhancement of different biological properties that is otherwise impossible to be achieved [10,11].

### 2.1. Physical and Chemical Synthesis

Capping agents and reducing agents are added separately during physical and chemical methods of nanoparticles’ fabrication. The capping agents are mostly added during synthesis of nanoparticles [8,12]. Polyethylene glycol (PEG), polyvinylpyrrolidone (PVP), polyvinyl alcohol (PVA), bovine serum albumin (BSA), ethylene diamine tetra acetic acid (EDTA), and chitosan are a few of the well-known examples of capping agents used in nanotechnology [13]. The purpose of any of the capping agent is to control the over-growth of nanoparticle clusters and inhibit them from being agglomerated. All of the above-mentioned capping agents have the capability to bring about significant physicochemical changes in nanoparticles, thereby ideally used for nanomedical purposes [14].

The capping agents should be employed for achieving the necessary physicochemical and functional properties in nanoparticles. They differ from one another on the basis of their functional groups and surface charge. The selection of capping agents for the purpose of capping is dependent on the desired size and morphology of nanoparticles besides their possible applicability [12]. Other aspects are also considered, including the type of functional groups, the hydrophobic or hydrophilic nature of the functional groups, and the charge on capping agents, carbon-chain/linker length, etc. A slight modification in any particular parameter can have a substantial impact on the overall nanoparticle properties [6].

Recently, the anticancer activity of Mg nanoparticles capped with PVA and tungsten oxide (WO_3_) capped PVP nanoparticles was reported by Selvam et al. [15] and Popov et al. [16], respectively. Antidiabetic potential of chitosan capped liposomes was determined in the diabetic mice model by Shalaby and El-Refaie [17]. Javed et al. [18,19] evaluated the antibacterial, antioxidant, antidiabetic, and cytotoxic activity of CuO nanoparticles capped with PEG, PVP, and chitosan nanoparticles under in vitro conditions. The antifungal potential of dodecanthiol capped Ag nanoparticles was obtained by Ferreira et al. [20].

### 2.2. Biological/Green Synthesis

The nanoparticles if prepared using plant, algal, fungal or bacterial extracts are posed to the enzymes and phytochemicals found in these extracts which function as potent reducing and capping agents. No capping agent is given from the outside environment in the case of biogenic synthesis of capped nanoparticles, instead they are provided naturally from organismal extracts. An organism or extract is selected for the fabrication of nanoparticles on the basis of its content of phytochemicals or enzymes, and its additional biomedical properties [21,22].

For instance, Fe_2_O_3_ nanoparticles fabricated using the *Rhus punjabensis* extract revealed tremendous anticancer, antibacterial, and antileishmanial effects [23]. The Ag nanoparticles prepared using a *Parkia speciosa* extract were shown to be antioxidant and antibacterial in nature [24]. The Ag nanoparticles synthesized using actinobacterial strain SF23 elucidated strong antibacterial and anticancer activity by Wypij et al. [25]. Similarly, a strong anticancer, antibacterial, and wound healing effect was produced by the Ag nanoparticles fabricated from the mycelial extract of endophytic fungus, *Talaromyces purpureogenus* [26]. The marine algae, Ecklonia cava-mediated Ag nanoparticle synthesis was reported by Venkatesan et al. [27]. These nanoparticles deciphered good antimicrobial, antioxidant, and anticancer activity.

It has been reported that the biological synthesis of nanoparticles offers more biocompatible, inexpensive, and eco-friendly approach that offers very lower toxicity and higher activity. However, the synthetic reactions and nanoparticles’ production mechanisms require deeper understanding by experimentation utilizing state-of-the-art technologies [28]. Since different types of bioresources are utilized for the green chemistry approach, every natural resource has its own scientific merits and demerits. Regarding bottlenecks, bacterial use offers difficult handling, downstream processing, and mass cultivation, fungi usage offers pathogenicity risk, while the green synthesis using algae and plants is relatively slow and time-taking process besides easy handling and availability of plants [29].

## 3. Effects of Capping Agents on Drug Delivery

**Figure 2:**
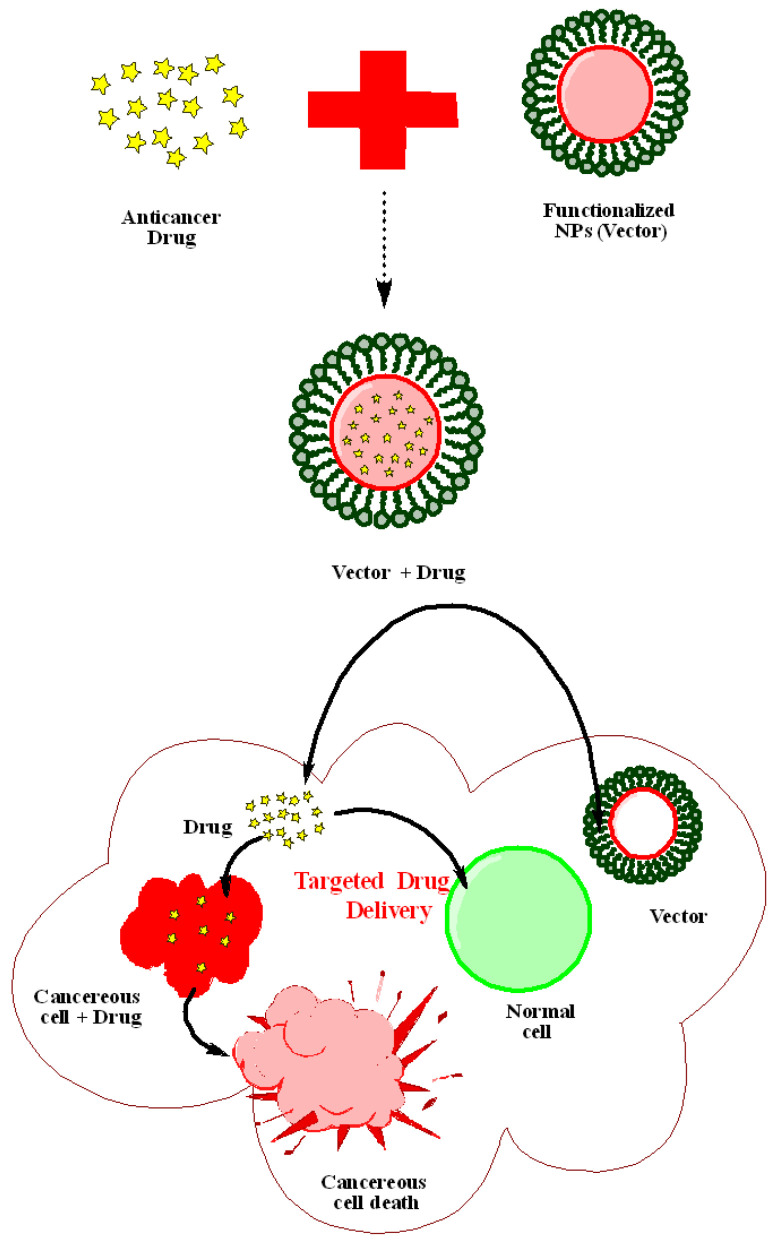
Simplified mechanism of drug delivery mediated by capped nanoparticles.

Nanotechnology has revolutionized each domain in which it has been used since its beginning. In the field of medicine, nanotechnology provides a wide range of tools that can be used to alter and improve traditional therapeutic and diagnostic approaches. Nanotechnology has a lot of potential in the area of medicine, leading to the development of new smart drug delivery systems that can administer drugs more effectively. With this, researchers’ goals are to develop effective support for the development of controlled release of nano-based devices that can serve as multifunctional delivery systems for encapsulating drug molecules, increasing their bioavailability, and resolving issues like poor water solubility and drug stability, and undesirable side effects. This goal is particularly appealing in the delivery of cancer-fighting cytotoxic drugs. Based on these concepts, a variety of nano-based devices have been developed for the encapsulation, transport, and release of antineoplastic drugs, using nanosystems such as dendrimers, polymeric nanoparticles, liposomes, lipid–polymer hybrid nanoparticles, and inorganic nanoparticles [30,31,32]. Figure 2 illustrates the mechanism of drug delivery of capped nanoparticles for cancer treatment.

In the same way, the development of controlled-release nano-devices that can remain stable and bioavailable for longer durations in the human body milieu is a goal that is being pursued. PEG has been frequently utilized in this arena. It is a hydrophilic polymer that is widely used in drug delivery formulations nowadays. Because of the hydrophilic ethylene glycol moieties, PEGylation of nanoparticles has been shown to enhance their solubility in buffer and serum. Furthermore, the inclusion of PEG groups on nanoparticle surfaces decreases the non-specific binding of nanoparticles to blood proteins and macrophages, resulting in the “stealth” behavior. Thereby, the blood circulation half-lives of PEG-containing nanocarriers have been increased, and passive targeting of cancer cells via the enhanced permeability and retention (EPR) effect may be improved [33]. Stabilization of colloidal nanoparticles with polymers like PEG has also been suggested as a way to extend their half-life in the blood and improve the selective delivery of these nanoparticles [34]. It is possible to extend the circulatory half-life of nanoparticles from minutes to hours or days by coating them with a neutral and hydrophilic substance such as PEG [35], PVA [36], and polysaccharides [37].

Many studies have recently shown that Au NPs may easily penetrate blood arteries and tissues into tumor sites, demonstrating that Au NPs are efficient drug carriers capable of minimizing cytotoxicity to nearby cells [38,39,40]. Brown et al. [38] reported the production of a platinum-tethered Au NPs fabricated with a thiolated PEG for enhanced anticancer drug administration in the lung epithelial cancer cell line (A549) as well as the colon cancer cell lines (HCT15, HT29, and HCT116), with the platinum molecule serving as an active component of oxaliplatin. Ramalingam et al. [41] produced PVP stabilized Au NPs coupled with doxorubicin (Dox@PVP-AuNPs) for the efficient treatment of human lung cancer cells. They found that many tumor suppressor genes are upregulated when Dox@PVP-AuNPs are used in cancer therapy. A biodegradable natural polymer like chitosan may be used to stabilize metallic nanoparticles, which can then be utilized to develop drug delivery systems. Because of its excellent interaction with and penetration across biological membranes, chitosan works as an effective adjuvant [39]. Chitosan is frequently utilized because of its excellent biocompatibility and non-toxicity. Chitosan is utilized in the preparation of Au NPs as a stabilizer [42] and a reducing agent [43]. In Au NPs, the electrostatic attraction between metal anions and protonated amine groups is due to the interaction between chitosan and anionic tetrachloroauric ions [44]. In a recent study, folic acid (FA) conjugated PVP-functionalized Au NPs loaded with curcumin were used to target breast cancer cells without damaging normal cells [45]. In another study, it was shown that curcumin’s cytotoxic effect was enhanced when it was linked to one of the side chains of water-soluble polymers, such as PEG, PVP, and chitosan, while the other side chain was coupled to Au NPs which also tackled the non-specificity and low solubility problems of curcumin [46].

Various polymers have been successfully used to decorate Ag NPs, which have since been used in a range of medical applications including antimicrobials and drug delivery. The majority of Ag NPs bio-conjugation research has mainly focused on antimicrobials [47]. Ravindra et al. [48] created a hydrogel containing curcumin-loaded Ag NPs and investigated their antibacterial properties as well as drug delivery applications. They found the controlled release of curcumin from modified Ag NPs in a regulated manner and were considerably more potent against *E. coli* than uncapped Ag NPs, suggesting that polymer-coated Ag NPs constitute an effective drug delivery platform. Coated Ag NPs have been proven to be effective antiviral agents in comparison to bare Ag NPs, and Ag NPs can function as a defensive nano-shield against viral infection [49]. Elechiguerra et al. [50] investigated the antiviral efficacy of Ag NPs capped with three different polymers: foamy carbon, PVP, and BSA against the human immunodeficiency virus type 1 (HIV-1). According to them, PVP and BSA coupled Ag NPs had lower inhibitory efficiency than foamy carbon Ag NPs. Antiviral efficacy of Ag NPs with PVP, BSA, and recombinant F protein (RF 412) against the respiratory syncytial virus (RSV) has been demonstrated by Sun et al. [51]. All of these conjugated Ag NPs were used in Hep-2 cell culture to fight RSV infection. When exposed to Ag NPs capped with BSA and RF 412, the RSV forms a non-specific attachment. However, PVP-capped Ag NPs showed a high affinity for binding to the viral surface and a strong interaction with the G proteins on the RSV envelope [51].

A surface modification or a capping agent capable of conjugating with therapeutic (drug) molecules [52] is needed to avoid magnetite nanoparticles from aggregating. Polymers or surfactants are commonly used in nanoparticle coatings because cross-linked polymers can inhibit coagulation and develop monodisperse particles [53]. For example, PVA can be employed to stabilize magnetite nanoparticles because of their hydrophilic characteristics and biodegradability [54]. Biomedical uses of PVA include biocompatibility, non-toxicity, non-carcinogenicity, non-immunogenicity, and inertness in bodily fluids [55]. Similarly, PVP as a polymeric carrier has been suggested for drug delivery in a variety of morphologies. In addition to allowing for a controlled drug release, PVP improves the bioavailability of poorly water-soluble drugs, protects the active component from environmental agents (pH, temperature, and oxygen), and masks undesirable smells and flavors [56].

Magnetite (Fe_3_O_4_) nanoparticles were coated with PVP by Rose et al. [57] to ensure magnetically directed drug delivery. The model anticancer drug, epirubicin hydrochloride, was then adsorbed onto the surfaces of both PVP-coated and uncoated nanoparticles. Compared to uncoated nanoparticles, the PVP coating decreased the aggregation of nanoparticles and resulted in a greater drug loading of 78%. PVP-coated nanoparticles demonstrated 81% growth suppression in breast cancer cells, proving their cancer-targeting potential. However, Ansari et al. [58] synthesized the PVA capped Cu_0.3_Zn_0.5_Mg_0.2_Fe_2_O_4_ nanoparticles using ibuprofen as a model drug. They proposed that these mixed nanoparticles could be applied as a new drug delivery system. EDTA capping is a kind of organic coating that has a wide range of uses in the chemical, pharmaceutical, and cosmetic sectors [59]. EDTA’s primary benefit in drug delivery systems is its capacity to serve as a chelating agent, which inhibits oxidation and radical reactions by chelating metal ions which are responsible for harmful effects on human bodies [60]. In addition to its electronegativity, EDTA may attract positive ions. This characteristic has been used in a variety of biological and chemical applications [61]. EDTA is utilized in medicine to improve kidney function [62] as well as in dentistry [63] and drug delivery systems [60]. Aghazadeh et al. [64] reported a new technique for the production of iron oxide nanoparticles (IONPs) capped by EDTA based on the one-pot electrodeposition method, and these NPs showed acceptable characteristics for application in the biomedical sector. The significant influence of different capping agents on nanoparticles for targeted drug delivery has been explained below (Table 1).

## 4. Effects of Capping Agents on Gene Delivery

Since the physicochemical properties of oligonucleotides and low molecular weight drugs are so dissimilar, different procedures to encapsulate these two unique payloads are usually required. Small-molecule drugs can be encased in nano-carriers through chemical conjugation, electrostatic contact, and hydrophobic force, whereas gene agents are typically compressed by the carriers via electrostatic force [89]. Gene delivery is a potential approach for treating disorders caused by aberrant gene expression, both inherited and acquired. It includes the use of vectors to deliver foreign genetic material to target cells, as genetic material delivery suffers from unanticipated degradation in the physiological environment. In research and clinical practice, two vectors are now used: viral and non-viral. A non-viral vector has been widely used in a broad range of gene delivery applications due to its simple and flexible chemistry as well as its cost-effectiveness despite their low efficacy. Polymeric systems (nanoparticles, micelles, and dendrimers), carbon nanotubes (CNTs), ceramic particles, liposomes, and metal nanoparticles (nanoparticles and nanorods) have all been exploited as carrier systems in the last decade [90,91]. Six cancer clinical trials are presently underway using nanoparticle-based small interfering RNA (siRNA) delivery; however, all nanoparticle-formulated siRNA delivery methods in clinical trials for cancer therapy are focused on liposomes or polymers [91]. Due to less biocompatibility, insufficient intracellular release, extracellular stability, nuclear delivery, and poor loading efficiency, the efficacy of these constructs is always minimal for clinical application.

Among many carrier systems, Au NPs are the most widely used metal nanoparticles for gene delivery and other biological applications, such as therapeutic delivery vehicles and diagnostics [92]. Au NPs can be easily functionalized with diverse moieties to improve internalization, and biocompatibility and their nanoscale optical characteristics allow them to be tracked intracellularly [93,94] Nucleic acids, such as siRNA, and oligonucleotides [95] can also be delivered via Au NPs [96]. To functionalize Au NPs with nucleic acids, a variety of techniques have been established. Under certain conditions, DNA functionalized nanoparticles can be made. Studies on the thermodynamics and kinetics of DNA conjugated with Au NPs have shown that ssDNA binds to Au NPs and then spreads throughout their surface [97]. Another study found that conjugating aptamers with Au NPs via hybridization processes on oligonucleotide functionalized Au NPs is a better method than directly conjugating aptamers with Au NPs. The primary advantage is that the integrity of aptamers is preserved, requiring fewer aptamers to complete the conjugation process. The detection of prostate cancer cells was accomplished using this method [98]. Yonezawa et al. [99] synthesized thiocholine-modified Au NPs that bonded to DNA and formed a fusion of wire-like structures across the DNA. Sandstrom et al. [100] demonstrated the ability to attach nucleic acids to Au NPs and Rosi et al. [101] used tetra thiol modified antisense oligonucleotides to bind to 13 nm Au NPs in a similar way. The ability to attach nucleic acids to nanoparticles opens the door to targeted gene delivery, which might result in genes coding for a specific protein being given to a cell that is either low in that protein or unable to create it [102].

**Figure 3:**
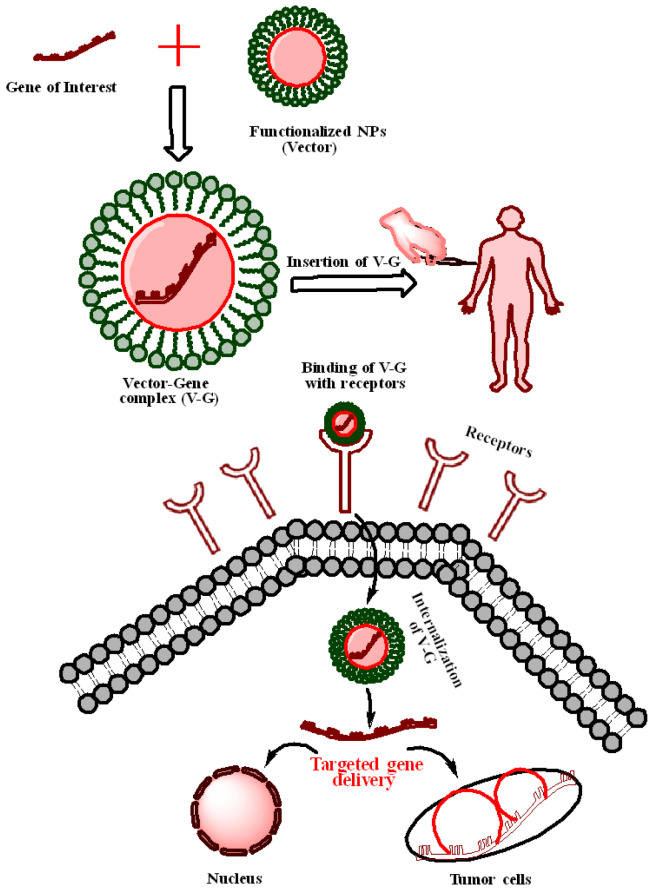
Possible mechanism of gene delivery mediated by capped nanoparticles.

By conjugating antimicrobial peptides with cationic Au NPs for gene delivery to mesenchymal stem cells, Peng and associates have used Au NPs for simultaneous antimicrobial and gene therapy [103]. Due to the possibility of treating many human diseases by delivering a functioning copy of a faulty gene or delivering siRNA, antisense oligonucleotides (ASOs), micro RNA (miRNA), and short hairpin RNA (shRNA) to cells, the delivery of therapeutic nucleic acids (TNAs) to cells has been a focus of high hopes [104]. TNAs must be protected from nuclease degradation in the optimal transfection reagent, allowing them to be released into the nucleus. One of the benefits of attaching nucleic acids to Au NPs’ surfaces is that the nucleic acid is shielded from nuclease degradation due to steric hindrance [105]. Au NPs are increasingly being employed in vitro and in vivo for gene therapy because of their low toxicity, high payload (owing to large specific surface area), quick endosomal escape, improved uptake, effective and selective gene silencing, and increased half-life [105,106]. Figure 3 explains the process of gene delivery of capped NPs for cancer treatment.

The efficacy of gold nano-conjugates coupled with oligonucleotides in gene therapy has also been proven [107]. Vinhas and colleagues found that Au NPs functionalized with an antisense oligonucleotide against BCR-ABL mRNA, a fusion mRNA that when translated produces a constitutively active tyrosine kinase that plays a key role in leukemogenesis, induce efficient silencing and an increase in K562 cell mortality [108]. Using Au NPs functionalized with chitosan, acylated chitosan, and chitosan oligosaccharide nano-conjugates, Abrica Gonzalez and colleagues investigated the effectiveness of DNA transfection in HEK293 cells. The chitosan oligosaccharide produced the best efficiency [109].

Bioconjugated Ag NPs have also been employed to treat a variety of ailments as innovative nano-carrier systems. In comparison to commercial transfection vectors, the DNA-decorated Ag NPs were shown to be unique nanocarriers that help to enhance nuclease stability, hybridization activity following cellular uptake, and photo release. Wang et al. [110] developed a procedure for making doxorubicin and FA-coated Ag NPs (FA-Ag NPs) for drug delivery. Soumya and Hela [111] also investigated the role of conjugated Ag NPs in cancer therapy. Ag NPs coupled with PEG were discovered to be effective nanocarriers for anticancer drugs.

Functionalization of IONPs with polyethyleneimine (PEI) has been utilized to increase internalization and lysosomal release [112]. In addition to this, a magnetic field boosts the effectiveness of DNA delivery by delivering nanoparticles through the cell compartments. The use of DNA-loaded magnetic iron nanoparticles in mitochondrial therapeutics aims to induce cell death by interacting with a mitochondrial translocation protein. The effect of magnetism and gene silencing techniques was examined using superparamagnetic iron oxide nanoparticles (SPION) by Kim and colleagues [113]. Using a magnetic field to transfer the carrier to an appropriate location increased the effectiveness of transfection and boosted the induction of the intrinsic apoptotic pathway, according to these researchers. For silencing gene therapy, iron nanoparticles can be used because of their small size and variable functionalization, which generates a net positive surface charge that boosts the effectiveness of siRNA. Fe_3_O_4_ nanoparticles were recently employed to target B-cell lymphoma2 (BCL2) in Ca922 oral cancer cells, and the combination with magneto therapy were able to enhance the gene silencing effect [112]. A significant impact produced by different capping agents on nanoparticles for gene delivery has been described below (Table 2).

## 5. Effects of Capping Agents in Bioimaging

Bioimaging deals with imaging or visualization of biological processes and structures. Plethora of tools and techniques have been designed to fulfill the requirements in different clinical and biological laboratories [121,122]. The bioimaging techniques including MRI, computed tomography (CT), positron emission tomography (PET), single positron emission computed tomography (SPECT), and ultrasound (US) have been employed for the detection and diagnosis of malfunctions or diseases. These techniques are actually non-invasive and some can give rise to high-resolution images of internal structures. Although in the majority of cases imaging probes can label organs and molecules of interest that offer improved visibility and further allow the procurement of more detailed and comprehensive structural and functional information [123,124]. This leads to the extensive use of imaging probes in research and disease diagnosis. The latest developments in imaging probes have prompted bioimaging at the molecular or subcellular level [125,126].

Mainly, organic and metal based organic compounds are currently used in imaging probes [127,128,129] at clinics whose usefulness is restricted due to its innate physiochemical characteristics, like fluorescent dyes employed in optical imaging exhibit photo bleaching, small stokes shifts, and have little or poor solubility in water [130,131], and contrast agents used in MRI made of Gd^3+^-chelates demonstrate weak contrast effect because of their low magnetic moment [131]. These small molecule-based probes also exhibit short circulation, retention, and imaging time which ultimately results in poor targeting competence and inadequate imaging augmentation [132]. Moreover, these molecules are also toxic which question their biocompatibility and use in biological applications. To overcome these limitations of contrast agents or imaging probes, various nanotechnological based substances like core-shell nanoparticles have been examined as potential contrast agents due to their increased biocompatibility and imaging time. For example, magnetic nanoparticles like SPIONs have been used as strong T2 MRI contrast agents, displaying enhanced sensitive detection over traditional Gd^3+^-based MRI contrast agents [133,134]. High X-ray attenuation of nanoparticles of high charge elements like gold [135,136] bismuth [137,138], and tantalum [139,140] have been explored as contrast agents for improved CT. Superior optical and chemical stability of QDs and their easily tunable emission wavelength has made them as robust fluorescent tags in optical imaging [141,142]. Up-conversion materials doped with rare earth metals such as NaYF4: Yb and Er nanocrystals can be excited in the near infrared region (NIR), then emit at a specific shorter wavelength, which makes penetration into thick biological tissues that are desirable in biological imaging easy, avoiding tissue’s auto-fluorescence. In spite of these advantages, these nanoparticles exhibit some shortcomings that avert their wide use in biomedical applications including magnetic artifacts of T2 MRI contrast agents, ultraviolet (UV) excitation of quantum dots (QDs) inducing tissue damage, and potential toxicity of QDs and rare earth-metal doped up-conversion nanomaterials [143,144,145,146].

**Figure 4:**
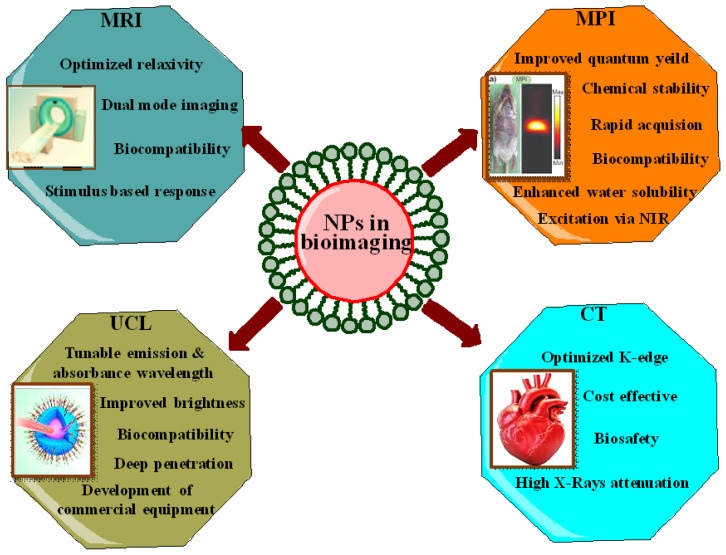
The capped nanoparticles used in various types of bioimaging.

In recent years, several attempts have been made to overcome the limitations associated with inorganic nanoparticles imaging probes. The most effective approach involves surface modification of nanoparticles through capping agents that offer better and improved biocompatibility. Furthermore, capping agents provide desired functionalities like stimuli responsiveness, targeted imaging and therapy [145]. For example, noble metal nanoclusters having less than 2 nm of dimensions hold unique fluorescent features which make them an attractive candidate for clinical applications [147,148]. Proteins, peptides, or small organic molecules are used as a template or capping agent in the synthesis of these noble metal nanoclusters which attribute biocompatibility, surface modification, and stability under irradiation, large stokes shift, and dimension dependent excitation and emission spectra, which offers different emission maximum from the visible to infrared wavelength regime as depicted in Figure 4 [149,150]. Besides this, there are various reports in the literature that demonstrate the effect of the capping agents on the nanomaterials used in bioimaging (Table 3).

## 6. Effects of Capping Agents in Biosensing

Recently, innovation in nanotechnology is leading to its applications in miscellaneous fields particularly in nanomedicine and nanobiotechnological based processes including drug delivery, new therapeutic modalities for imaging, prevention or cure of deadly diseases, biological sensing and detection, etc. [176]. Detection or sensing of wide range of molecules at a lower or trace amount with high specificity has prompted advances in designing new devices which incorporate advanced biochemical nanomaterials are known as nanobiosensors. Biosensors have attained significant research interest throughout the world due to their simplest incorporation into disposable polymers for various applications in agriculture, food, environment, and biochemical and clinical analysis due to their easy, economical, quick, sensitive, specific, on work, online, and real-time detection. Therefore, biosensors with high sensitivity provide an exceptional analytical tool in different fields [177,178].

**Figure 5:**
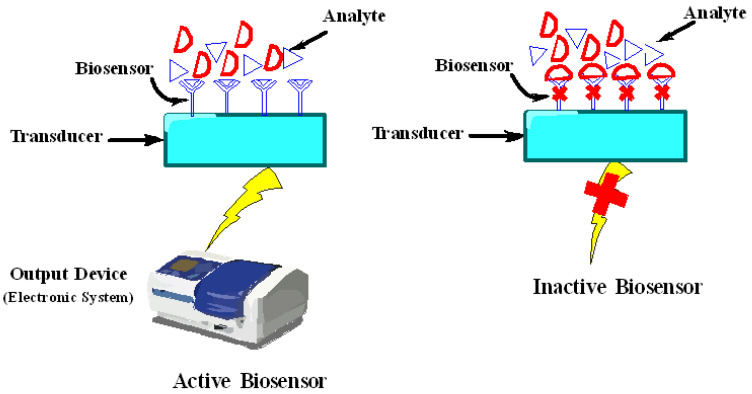
Basic components of biosensors.

Nanomaterials have allowed the development of ultrasensitive biosensors due to their intrinsic characteristics like large surface-to-volume ratio which helps in immobilizing variety of biomolecules encompassing electric and catalytic properties which in turn make the direct electron transfer among the electrodes and from the active sites of biological molecules. Technically, biosensors comprise of a biosensing material/receptor and a transducer which can detect chemical or biological substance upon specific binding (Figure 5). Biosensing material is actually a biological material like cells, tissues, organelles, enzymes, antibodies, nucleic acid probes, etc. that can selectively recognize the target analyte, while transducer is a device like electrochemical, optical, piezoelectric, thermal, and magnetic which can quantitatively screen or monitor a biochemical reaction. Numerous types of nanomaterials including paramagnetic nanoparticles [179,180], QDs [181,182], carbon nanotubes (CNTs) [183,184], nanoshells [185], nanowires, nanoneedles, nanosheets, nanotubes, nanorods, nanobelts, etc., are actively used in sensing [7,186]. Nanomaterials, either used as they are or in conjugation with bioactive substances, become prominent due to their ability to bring an appropriate platform for developing high performance biosensors with unique properties [187].

**Figure 6:**
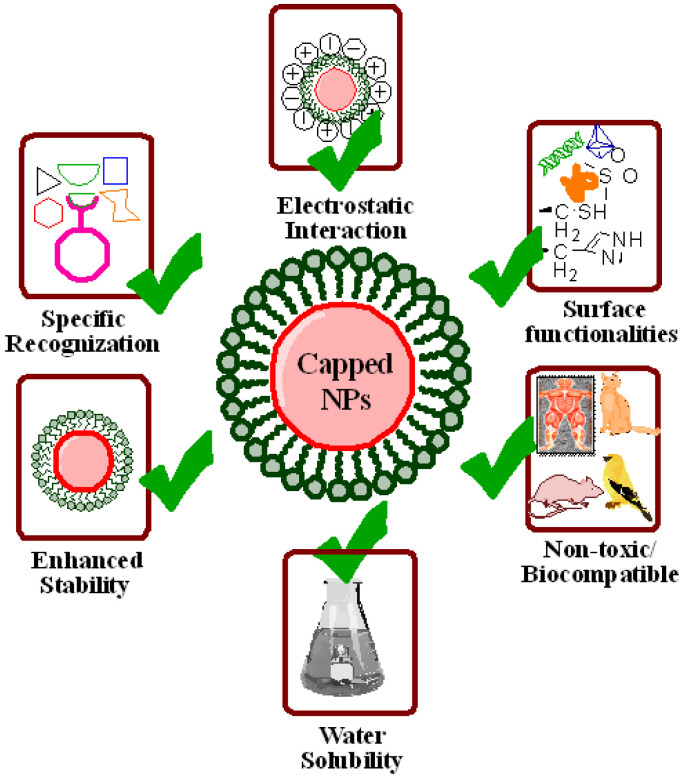
The capped nanoparticles used in biosensing.

Biocompatible nanomaterials are ideal for drug delivery, imaging, labelling, and biosensing. Furthermore, nanomaterials used as biosensors must be chemically stout to endure diverse conditions like display nominal perturbation of the probed system, e.g., lower to no toxicity, and generate sharp and switchable phenomena to incident light, producing a strong signal change during interaction with analyte. These problems can be resolved through covering or capping of nanoparticles’ surfaces which act as a barrier between the core and its environment. Moreover, they also help in further conjugation of nanomaterials’ surface, protection against moisture or chemical reactions, extend nanomaterials’ shelf life, and various shapes, dimensions, and geometry of nanomaterials can be achieved along with dispersion in different solvents, etc. [188]. For this, capping agents are the critical molecules to attain the preferred physicochemical properties and functionality of the nanoparticles as shown in Figure 6 [189]. The vast majority of biomolecules and ions in physiological fluids offer a hostile environment for nanomaterials; for this, capping ligands must afford effective protection. On the other side, thickness of capping layer is also critical for the working of biosensors. In biosensing, nanomaterials’ signal modulation in response to analyte interaction is generally directed by distance dependent interactions across the capping layer where a thinner layer leads to stronger signal modulation. These interactions consist of resonance energy transfer, where energy transfer is carried out by non-radiative dipole–dipole coupling across space, mostly up to 10 nm, and localized surface plasmon resonance (LSPR) field perturbation in which substances entering the LSPR field change the refractive index of sensing volume surrounding the plasmonic nanoparticles which are generally up to 30 nm from surface. This sets a check on capping layer, i.e., it must be greatly protected and compact. On the other hand, thinner capping layers are less protective against particle’s aggregation, surface degradation, and non-specific adsorption. Currently, the most effective surface capping techniques utilize custom molecules with modular combinations of low molecular weight constituents which imparts a range of useful characteristics to the nanomaterials [190]. Furthermore, better control over polymerization and surface coordination results in generating superb polymer capping agents [191]. Although precise nature of interactions between nanomaterials and environment has not been clearly interpreted, but there are various processes that create an obvious effect upon the working of biosensors [192].

The charged nanoparticles fascinate a large number of ions which can vary the local environment by inducing ionic and pH gradients, which ultimately affect mechanisms of biosensors and conformation of biological molecules. Hence, nanomaterials are capped with some suitable substances like chitosan capped magnetic nanoparticles exhibit no toxicity, enhanced stability, surface modification, specific recognition, and induce electrostatic interaction as shown in Figure 5 [193,194]. Along with this, there are various reports available in the literature that show the effect of capping agents in biosensing. Some of them are listed here. Nancomposites formed by poly (diallyldimethylammonium chloride) capped gold nanoparticles (Au NPs) with the functionalized graphene (G) multiwalled carbon nanotubes (MWCNTs) were used as a glucose sensor. Characterization techniques revealed that this composite material was able to immobilize more enzyme molecules resulting in quickest and direct electron transfer between redox sites of enzyme and electrode [195]. The copper-zinc superoxide dismutase (SOD) was immobilized on the surface of biocomposite film made up of the AuNP-chitosan-ionic liquid used as the anion sensing biosensor. Results demonstrated that this nanosensor was highly sensitive towards a superoxide anion with the lowest detection limit of 1.7 nm [196]. Another study depicted the fabrication of chitosan capped Au NPs for sensing carcinoembryonic antigen (CEA) and alpha-fetoprotein (AFP) as an electrochemical immunosensor [197]. The differential pulse voltammetry (DPV) method was employed for detection of metal ions. Moreover, a nanocomplex comprised of Au NPs, graphene oxide (GO), and EDTA was designed for the detection of dopamine in living cells [198]. Folic acid functionalized Au NPs, and a ferrocene based cytosensor was devised for the detection of cancerous cells. Functionalized Au NPs accelerated electron transfer between signal indicator and the electrode, and also facilitated accumulation of more ferrocenes due to nanodimensions, hence improving overall sensitivity [199]. Thioglycolic acid (TGA) and L-cysteine capped cadmium telluride (Cd Te) quantum dots (QDs) were constructed and employed as an optical sensor for the detection of Hg^2+^ ions even at a very low concentration range of nanomolar and picomolar. Results demonstrated that TGA capped Cd Te QDs depicted a linear response towards Hg^2+^ ions in the concentration ranges from 1.25 to 10 nM, while L-cysteine capped Cd Te QDs showed a sensitivity of 6 × 109 M^−1^ with a linear coefficient of 0.99 and detection limit of 2.7 pM ranges from 5 to 25 pM of Hg^2+^ ions [200]. Malic acid (MA) and polyvinylpyrrolidone (PVP) capped Ag NPs were employed as a biosensor for glucose detection. Results elucidated that PVP capped Ag NPs produced an efficiently stable potentiometric response with enhanced stability, sensitivity, immobilization, and absorbance rate as compared to bare Ag NPs and MA capped Ag NPs [201]. In another study, Au NPs were capped with β-cyclodextrin whose surface was designed for a tyrosinase (Tyr)-based sensor. Furthermore, an Au NPs-β-cyclodextrin complex was linked with graphite electrode where Tyr was immobilized and employed as a drug inhibition platform where catechol acted as a substrate. The detection limit of this biosensor was 0.42 μM with a sensitivity of 2.094 μA.μM^−1^ cm^−2^. This biosensor acted as an ideal for detecting the Tyr inhibition through electrochemical methods [202].

Immunosensors for carbohydrate antigen (CA 15-3) were developed through grafting of Ru (ll) luminophore and poly (ethylenimine) (PEI) on palladium nanocages (Pd NCs). The Pd NCs, due to high surface area, good electrocatalysis, and special structure with porous walls and hollow interior, offer to load ample PEI, and decoration of Ru (bpy) 2(5-NH2-1,10Phen) Cl2. Graphene functionalized Au NPs were used as a substrate to immobilize the first antibody. These immunosensors displayed a linear range of 0.01–120 Uml^−1^ with a lower detection limit of 0.003 Uml^−1^ [203]. In another report, AFP detection was done through a solid state Ru (bpy) 32-ECL sandwiched biosensor. In this, reduced graphene oxide (RGO) was functionalized with PEI forming PEI-rGO which immobilized Au NPs incorporated with poly (amidoamine) (PAMAM) as a second antibody carrier and Nafion-Ru-PtNP acted as a first antibody platform and the ECL substrate. Weak conductivity of PAMAMA was improved through Au NPs [204]. A bioenzymatic glucose biosensor was made by doping of Au NPs on chitosan membrane as a biocompatible enzyme system which improved a luminol chemo-luminiscence (CL) reaction. The enzyme glucose oxidase (GOD) oxidizes glucose producing hydrogen peroxide (H_2_O_2_) and gluconolacton, and horseshoe radish peroxidase (HRP) oxidizes luminol through H_2_O_2_ resulting in enhanced CL signal. This enhancement is due to the Au NPs that act as mediator in electron transfer and help HRP to come back to its reduced form [205]. Gold nanowires fabricated through nanopore polycarbonate (PC) membrane were dispersed into chitosan (CHIT) further immobilized onto the surface of glassy carbon electrode. This electrode enabled fast and sensitive detection of low potential H_2_O_2_. Adsorption of GOD on the surface of nanowires was used to develop a glucose biosensor [206,207]. Likewise, platinum nanowires (Pt NWs) and CNTs have been solubilized into CHIT and produced PtNW/CNT/CHIT film. This film modified electrode that provided a marked reduction in the overvoltage for the H_2_O_2_ and was an excellent amperometric sensor for H_2_O_2_ at −0.1 V over a wide range of concentrations with 260 μAmM^−^^1^cm^−^^2^ recorded sensitivity. By connecting GOD, an amplified glucose biosensor was established, which demonstrated selective measurement of glucose at −0.1 V. The application of polypyrrole-coated GOD nanoparticles (GOx/Ppy) in an electrochemical biosensor device demonstrated a 10 times increase of Km as compared to the native GOx [208].

Another investigation reported the use of chitosan functionalized CNTs as a biosensor for salmon sperm’s DNA detection via methylene blue (MB) as a DNA indicator. A low detection limit of salmon sperm’s DNA was around 0.252 nM, and no noise was detected even in the presence of 5 µg/mL human serum albumin (HSA). A differential pulse voltametric signal of MB was linear over the wide range of concentration (0.5–20 nM) of sperm’s DNA [209]. In another approach, graphene was functionalized with positively charged poly (1-vinyl-3-butylimidazolium bromide)-graphene which successfully immobilized negatively charged GOD onto the poly (ViBuIm+Br−)-G forming a GOD/poly (ViBuIm+Br−)-G/glassy carbon (GC) electrode under mild conditions. This electrode revealed marked sensitivity, along with a wide linear range and significant stability in glucose detection [210]. Water soluble and biocompatible poly-l-lysine (PLL) functionalized graphene sheets were fabricated that played a vital role as connectors to attach other bioactive molecules [211] while water dispersed polyvinylpyrrolidone graphene with a good electrochemical reduction towards O_2_ and H_2_O_2_ was prepared by another group. The polyvinylpyrrolidone graphene further functionalized with a polyethyleneimine GOD electrochemical biosensor. This composite based material provided the direct electron transfer of GOD with sustainable bioactivity, and demonstrated the potential application of novel glucose biosensors development with linear glucose response up to 14 mM [212].

Furthermore, a study showed the effect of capping agents, i.e., PEG, EDTA, PVP, and PVA on the size of Ag NPs and their potential application as a biosensor. Results demonstrated that, among all capping agents, PVA capped Ag NPs were of a smaller size, i.e., around 31 nm and 27 nm, obtained through XRD and TEM micrographs. These PVA capped Ag NPs were further explored for H_2_O_2_ sensing through an LSPR. This sensor effectively sensed H_2_O_2_ even at a low of 10^−7^ M [14]. Another study documented PVP-capped Ag NPs based on an LSPR based optical biosensor for the detection of H_2_O_2_ with a detection limit of 1 nM [213], while a polysaccharide capped Ag NPs based optical fiber nanosensor was constructed which detected H_2_O_2_ in a wide concentration range of 10^−2^ to 10^−6^ M [214]. A zinc sulfide quantum dot (ZSQT) capped with 2-mercaptoethanol (2-ME) was used as an optical sensor for the detection of cyanide ions with a detection limit of 1.7 × 10^− 7^ M at a pH of 11. The as prepared nanosensor exhibited obvious sensitivity for cyanide ions over other anions [215]. These reports stated the importance of surface capped nanoparticles in biosensing with improved outcomes as compared to naked nanoparticles.

## 7. Conclusions and Future Directions

Capping agents make the size of nanoparticles reduced, morphology and surface chemistry altered, and compactness and stability enhanced. The stabilizers chosen for capping of colloidal nanoparticles are biocompatible, non-toxic, and biodegradable. Capped nanoparticles possess substantial potential to design smart drug delivery systems that could ensure targeted delivery as well as slow and sustained release of drugs in the tumor microenvironment. Such nanoparticles have also shown unprecedented outcomes in the areas of gene delivery, bioimaging, and biosensing. The major roles of capping agents in all of these applications have been observed to be: (1) the regulation of growth of nanoparticles to obtain their controlled size and shape, and (2) the prevention of agglomeration of nanoparticles by reducing their surface energy so that they do not bind with one another, and rather remain intact. In addition, this strategy holds promise in overcoming concerns about nanosafety, and offering opportunities for progress in nanomedicine and other related areas but still ample work is required to fill the existing research gaps.

Different capping agents possess multifunctional properties and have been proven to play an integral role in improving the characteristics of nanoparticles in aspects that make them highly suitable to be employed in various fields of nanomedicine. However, choosing a suitable surface capping agent for the rational design and fabrication of nanoparticles for engineering desired progress in nanomedicine is indeed a big hurdle. Moreover, to perform highly optimized reproducible experiments regarding fabrication of size/shape-controlled nanoparticles seems very challenging for now. For instance, the majority of findings in the field of nanotechnology are still confined to the laboratory scale, owing to the nanoparticles’ low stability in real-world samples or environments, substantial batch-to-batch differences, and lack of multifunctionality resulting in anomalies in overall performance. These issues can be reduced or avoided by using appropriate capping agents on the surface of nanoparticles.

Though there is experimental evidence from literature about the remarkable effects of capping agents, analyzing the pure effect of capping agent (isolating from other factors) in a particular nanomedicine approach remains a challenge. The advanced characterization approaches should be devised to precisely quantify the adsorption of capping agent in a capped nanocomposite. Hence, several questions would be answered and issues resolved about synthesis process by the comprehensive interpretation of characterization of capped nanomaterials. In addition, the significant role of capping agents in a particular biological phenomenon should be elucidated in detail by undergoing both in vitro and in vivo experiments. This is how the main purpose of capping agents, which is to get controlled beneficial effect, could be achieved. In addition, extensive toxicity assessments are required because, if remained unnoticed, they will lead to serious consequences. Thus, the fate of capped nanomaterials designed by facile surface modification should be efficiently determined by future studies.

## Figures and Tables

**Table 1:** Effects of different capping agents of nanoparticles used in drug delivery.

Sr. No.	Nanoparticles (NPs)	Capping Agents	Drug	Targeting Disease/Cell Line Type	Mechanism of Action/Effect	Reference
1.	Ag NPs	PVA & Chitosan	Naproxen	Saos-2 cells	Strong response of Saos-2 cells with a higher level of adhesion, proliferation, and mineralization	[65]
2.	Ag NPs	PVA	DOX, Curcumin	Bacillus cereus, *E. coli*	Significant antibacterial activity	[66]
3.	Ag NPs	PVA, PVP	PVA-Ag NPs, PVP-Ag NPs	Skin wound	PVA-Ag NPs: Exhibit a dominant antibacterial efficacy and showed positive effects through their anti-inflammatory and angiogenic properties, with a nearly 95% healing effect within 9 days; PVP-Ag NPs: Potential antimicrobial efficacy and wound healing properties	[67]
4.	Ag NPs	PEG	I-131 radionuclide	WI-38 cells, solid tumor sarcoma bearing mice	High in-vitro and in-vivo stability, with no cytotoxic effect on normal cells at a lower concentration, high radioactivity accumulation in tumor tissues of mice	[68]
5.	Ag NPs	PVA/PVP/Pectin	Mafenide acetate	Skin wound	Remarkable effect on wound healing	[69]
6.	Ag NPs	Chitosan	Ag-Chitosan	Skin wound	Accelerated the healing of a burn wound by decreasing the inflammatory reaction; subsequently, decreasing the duration of the repair phase	[70]
7.	Fe_3_O_4_ NPs	PVA, SA, BSA	DOX	HepG2, L02 cell lines	Fe_3_O_4_-SA-DOX-PVA-BSA toxic to HepG2 cell lines and non-toxic to L02 cell lines	[71]
8.	Fe_3_O_4_ NPs	EDTA	Imatinib	Bone marrow cell line (K562)	Drug loaded NPs display lower liver accumulation compared to a bared drug, prolonged circulation time	[72]
9.	MnFe_2_O_4_ NPs	PVP	DOX	HeLa Cells	No cytotoxicity of PVP-coated MnFe_2_O_4_ nanoparticles. Controlled drug delivery with pH-dependent release behavior.	[73]
10.	PLGA NPs	Chitosan, PEG and Dextran	Curcumin	Breast cancer cells (MCF-7)	Effective in arresting cancer cell growth, induce apoptosis	[74]
11.	Mesoporous silica nanoparticles (MS NPs)	PEG	DOX	HeLa cells	Decrease in cancer cell viability	[33]
12.	MS NPs	PEGylated polyaminoacids	Celastrol (CST)	Cancer cells, and SCC-7 xenograft tumor-bearing mice	CMSN-PEG exhibited high in vitro cytotoxicity in different cancer cells, effectively used as a mitochondrial targeting system for efficient inhibition of solid tumors	[75]
13.	MNs loaded with PB NPs	PVA/PVP	Metformin	Skin	The effective decline in the BGLs of diabetic rats.	[76]
14.	Au NPs	PAMAM–COOH (G4)	DOX	Cancer	Enhanced permeation and retention (EPR) mediated drug targeting followed by the lysosomal drug release	[77]
15.	FeO NPs	PAMAM–NH2 (G4)	3,4-difluorobenz ylidene- curcumin	SKOV3 cells	Multivalent theranostic nanoparticles for simultaneous imaging and precise cancer cell targeting	[78]
16.	Au NPs	BSA	Methotrexate (MTX)	MCF-7	Inhibitory action on the growth of MCF-7 cell line induces apoptosis	[79]
17.	MS NPs	Peptide coated Au NPs	DOX	U87 MG cells and HEK 293 cells	NPs-mediated apoptosis of αvβ3 integrin over-expressing cancer cells	[80]
18.	Fe_3_O_4_ NC (nano-composite)	PAH/PSS	DOX	A549 cell lines	pH-responsive drug release and higher cytotoxicity towards human lung cancer (A549) cells in vitro in a dose-dependent manner	[81]
19.	Ultrasmall iron oxide nanoparticles (USIONPs)	Tannic acid (TA) and Quinic acid (QA)	Quinic acid (QA) and its derivatives	U87 cells and metastatic (MDA-MB-231Br cells)	Higher cellular uptake of QA-coated USIONPs compared to TA-coated USIONPs	[82]
20.	Ag NPs	Aesculus hippocastanum (horse chestnut)	Aqueous A. hippocastanum leaf extract, resveratrol	Bacterial agents, in vitro drug release	Significant antioxidant and antimicrobial activities, drug release from AgNPs exhibited pH dependency; the release was significant (45.6%) under acidic conditions (pH 5.2)	[83]
21.	Au & Ag NPs	B. monosperma (BM) leaf extract	DOX	B16F10 & MCF-7 cancer cells	Significant inhibition of cell proliferation in a dose-dependent manner (0.06–0.25 μM w. r. t DOX)	[84]
22.	PD-FeO NPs	CS, PVA	Leaf extract of Pinus densiflora (PD)	Diabetic and anemia-associated diabetic wounds	Enhanced cell proliferation and augmented angiogenesis, leading to wound contraction and reduction in cytotoxicity	[85]
23.	CeO_2_ NPs	CS/PVA	Zingiber officinale extract	Human dermal fibroblasts cells	Significantly decreased wound infections without the use of antibiotics	[86]
24.	ZnO NPs	Chitosan	Camellia sinensis/Paclitaxel	MCF-7	High cytotoxic effect on the breast cancer cell line	[87]
25.	α-Fe_2_O_3_ NPs	Nepeta cataria leaves extract	Doxorubicin	Melanoma cell line (A375)	Significant cytotoxic effect against the melanoma cancer cell line	[88]

Au NPs (gold nanoparticles); Ag NPs (silver nanoparticles); PLGA NPs (poly(lactic co-glycolic acid);* MNs (microneedles); *PB NPs* (perssian blue nanoparticles); CeO_2_-NPs (cerium oxide nanoparticles);* Saos-2 (Sarcoma osteogenic); DOX (Doxorubicin); WI-38 cells (Human lung fibroblast normal cell line); ROS (Reactive oxygen species); SA (Sodium alginate); BSA (Bovine serum albumin); HepG2 (Human cancer liver cell lines (hepatocellular carcinoma); and L02 (Human normal liver cell lines (hepatocytes); EDTA (Ethylene diamine tetra acetic acid); K562 (Bone marrow cell line); HMTA (Hexa methylene tetramine); HepG2, ATCC ^®^ HB-8065™ cell culture (Human hepatocellular carcinoma culture); MCF-7 (Breast cancer cells); A20 murine B-cell lymphoma cells; SCC7 (murine squamous cell carcinoma cell line); MNs (Near-infrared light triggered microneedles); PB (Perussian blue); HCT15, HT29, and HCT116 (colon cancer cell lines); A549 (human lung cancer cell lines); PAMAM–COOH (G4), PAMAM–NH2 (G4) (Poly amidoamine dendrimers); SKOV3 cells (Ovarian cancer cell lines); U87 MG cells (Human glioblastoma); HEK 293 cells (Human embryonic kidney cells); PSS (Sodium poly (styrene sulfonate)); PAH (Polycation Poly (allylamine hydrochloride)); HMBPs (Heavy metal binding proteins); USIONPs (Ultra smart iron oxide nanoparticles; SPIONs (Super paramagnetic iron oxide nanoparticles); MDA-MB-231Br cells (metastatic breast cancer cell lines); B16F10 (murine melanoma cell line).

**Table 2:** Effects of nanoparticles fabricated with different capping agents used in gene delivery.

Sr. No.	Nanoparticles (NPs)	Description/Capping Agent	Disease/Cell Lines	In Vitro/In Vivo Implication	Reference
1.	Au NPs	Synthesis of Au NPs capped with l-cysteine methyl ester hydrochloride conjugated to PEG	Hep G2, Caco-2, B16F10, and CT26	Bioengineered Au NPs with different sizes, shapes, structures, chemistry, and synthetic strategies have shown potential to enhance siRNA delivery in vitro and in vivo	[114]
3.	Au NPs	Linalool-loaded glutathione-modified Au NPs conjugated with CALNN peptide	SKOV-3	LG and LGC were selectively toxic in cancer cells and induced apoptosis by activating caspase-8, the p53 protein, and various proteins involved in apoptosis.	[115]
5.	Au NPs	Central core Au NPs encapsulated by a layer of DNA-capped QDs used	A pair of human ovarian carcinoma cell lines-A2780, and DOX-resistant cell line A2780 ADR	Programmable hybrid nanostructures engage with the target MRP1 mRNA, reduces the MRP1 expression, results in a detectable turn-on fluorescence signal, and Dox release. The Dox-anti-MRP1 hybrid is significantly more cytotoxic against MDR cancer cells.	[116]
6.	Au NPs	Au NPs fabricated with L-Cystine methyl ester hydrochloride as a capping agent, then loaded with plasmid DNA encoded p53 gene	WI38 and A549	The high percentage of cell viability in WI 38 proved the safety of L-cysteine methyl ester functionalized Au NPs. Additionally, the apoptotic effect due to the expression of p53 gene loaded on Au NPs was only prominent in A549.	[117]
7.	Au NPs	Doxorubicin loaded oligonucleotides (ONTs) attached to Au NPs (DOA)	SW480 and a xenograft mouse model	Successful cellular uptake of DOA by SW480, with significant cytotoxicity at reasonably low concentrations. In vivo, DOA could significantly suppress cancer growth in a mouse xenograft than free DOX	[118]
8.	Au nanosphere	Gold nanosphere coated with poly(ethylenimine) (PEI), conjugated with the targeting ligand anisamide (Au-PEI-AA)	PC3 prostate cancer cells	Au-PEI-AA mediated siRNA uptake into PC3 prostate cancer cells via binding to the sigma receptor, anisamide-labeled Au NPs can target the sigma receptor	[119]
9.	IONPs	Iron Oxide NPs to deliver siRNA targeting BCL-2 in oral cancer cells	Ca9-22 cell line	Reduced cell viability and relative cell migration in Ca9-22 cell line	[112]
10.	Au NPs	Biosynthesis of Au NPs using cold and hot sclerotium of *Lignosus rhinocerotis*, capped with chitosan	Human dermal fibroblasts (HDF)	DsiRNA-AuNPs incorporated into thermo-responsive pluronic gels demonstrated high cell viability, proliferation, and cell migration rate via in vitro cultured cells of HDF, indicating their non-cytotoxicity and wound healing properties	[120]

MRP1-Drug resistance factor; QDs (Quantum dots); DsiRNA (Dicer subtract small interfering RNA), DOX (doxorubicin); Hep G2 (human hepatocellular carcinoma cell line); Caco-2 (human colorectal adenocarcinoma cell line); PC-3 (human prostate carcinoma cell line); B16F10 (mouse melanoma cell line); CT26 (mouse colon carcinoma cell line); ovarian cancer cell line (SKOV-3); linalool–GNP (LG) and LGC (linalool–GNP–CALNN peptide); WI38 (healthy lung cells); A549 (cancerous lung cells); (SW480) colon cancer cell line; (Ca9-22) oral cancer cells.

**Table 3:** Effects of capping agents of nanoparticles and their possible uses in bioimaging.

Sr. No.	Nanoparticles (NPs)	Capping Agent	Effect of Capping Agent	Bioimaging Application	Reference
1.	CdS QDs	Dextrin	Reduced toxicity of innate cadmium sulfide (CdS)	Used as fluorescent agent in in vitro and in vivo studies where maximum fluorescence was observed in kidney, liver, and brain	[151]
2.	Au NPs & DTA	Polyamidoamine (PAMAM) dendrimer	Improved X-ray attenuation, stability, biocompatibility, and enhanced blood circulation time	CT Imaging	[152]
3.	Bimetallic Au-Ag NPs	Folic acid (FA)-modified PAMAM dendrimer	25% higher X-ray attenuation than Omnipaque. In in vitro better CT imaging of cancer cells overexpressing FA receptors showed 2.3 to 2.7-fold higher uptake than the cells possessing low level of FA expression.	In vitro CT imaging in cancer cells	[153]
4.	Ag NPs	Acetylated-PAMAM dendrimer	Extended blood circulation time led to prolonged enhancement	X-ray CT contrast agent	[154]
5.	Au NPs	Acetylated-PAMAM dendrimer	Improved biocompatibility, 1.6 times higher X-ray attenuation compared to Omnipaque, specific targeting through receptor-mediated endocytosis	In vivo CT imaging	[155]
6.	Au NPs	Thiolated PEG & pluronic triblock copolymer (PEO–PPO–PEO)	Improved colloidal and optical stability and biocompatibility	Used as scattering probes for dark-field imaging of cancer cells under both in vitro and in vivo conditions	[156]
7.	Gd_2_O_3_ & PMNPs	Diethylene glycol polymer & Liposomes	No in vitro cytotoxic effects, sensitive contrast agent	MRI contrast agent and marker for cell tracking	[157]
8.	IONPs	Dextran	Biocompatible, superior T2 relaxation rate and high relaxivities led to clear distinguished signal imaging intensity of specific organ, tumor, and whole-body	MRI contrast agents	[158]
9.	Au NPs	Poly di(carboxylatophenoxy)phosphazene	Biocompatible and biodegradable	Can be used as contrast agents for photoacoustic imaging	[159]
10.	INPs	PEG	Biocompatible, extended high contrast vascular imaging and stability, selectively accumulated in tumor	Vascular and tumor imaging by Micro-CT	[160]
11.	Iodine-131 labeled Au NPs	Polyethyleneimine (PEI)	Improved X-ray attenuation coefficient, colloidal stability, cytocompatibility, and radiochemical stability in vitro	Single-photon emission computed tomography/computed tomography (SPECT/CT) imaging and radionuclide therapy	[161]
12.	Bi_2_S_3_ NPs & QDs	PEG-phospholipid bilayer	Enhanced CT contrast and fluorescence imaging capability, longer circulation time (>4 h) than iobitridol, biocompatibility, and safety.	Used for combined CT/fluorescence imaging	[162]
13.	Radioactive iodide-124 labeled Au NPs	PEG	Non-toxic, high stability, and sensitivity in various pH, serum, and in vivo conditions	In vivo tumor imaging through combined positron emission tomography and cerenkov luminescent imaging (PET/CLI).	[163]
14.	CuS [c(RGDfK)]	PEG	High efficacy and minimal side effects	Promising platform for image guided ablation therapy	[164]
15.	Au NPs	Glycol-chitosan	Simplest nanocomposite did not require antibodies or complex surface modification	Photoacoustic contrast agent	[165]
16.	Silica NPs	PEG & doping with cyanine 5.5 (Cy5.5) & cyanine 7 (Cy7) dyes	High colloidal stability in water and in biological environment, with absorption and fluorescence emission in the NIR field	Used to achieve optical and photoacoustic imaging	[166]
17.	Au NPs	Poly(perylene diimide) (PPDI) & PEG	Greater photothermal effect and a stronger photoacoustic signal	Used as photoacoustic (PA) agents under in vivo imaging and therapeutic evaluation	[167]
18.	Plectin-SPION-Cy7 or SPION-Cy7	DSPE-PEG-NH_2_	Highly accumulated in tumor, MIAPaCa2 and XPA-1 carcinoma cells but not in normal pancreatic tissues, liver, and kidney	Optical imaging and MRI	[168]
19.	Curcumin-Ag NPs complex	Polyvinylpyrrolidone (PVP)	Enhanced water solubility and bioavailability in a biological system without effecting its therapeutic potential. Fluorescence efficiency in cancer cellular medium is ∼2.37 times higher	Used as fluorescent probe in CT imaging	[169]
20.	Au NPs doped with silver	Gelatin	Improved stability, quantum yield, and fluorescence lifetime. Remarkable biocompatibility	Promising approach for imaging in a challenging tissue as skin	[170]
21.	MoO_3_ mixed with optoelectrochemically active dye complex (Ru(II))	Chitosan (CS)	Biocompatible	Used in intracellular imaging	[171]
22.	Au NPs	*Zinnia elegans* plant extract	Highly biocompatible and do not use any targeted ligand	Used as imaging agent in NIR region	[172]
23.	Ag NPs	4-mercaptobenzoic acid-capped	Enhanced fluorescent brightness, improved photostability, and low cytotoxicity	Used for simultaneous cellular imaging and photodynamic therapy	[173]
24.	N-doped fluorescent Si NPs with an ultra-high quantum yield	EDTA-2Na	Water dispersibility, higher stability, and biocompatibility	Used in cellular imaging	[174]
25.	Carboxylated PPy-NPs	Folic acid functionalized carbon dots	Photostability, specific targeting, biocompatible	Used as PTT imaging agent	[175]

Gold nanoparticles (Au NPs); cadmium sulfide quantum dots (CdS QDs); radiodense iodine-containing compound (DTA); silver nanoparticles (Ag NPs); gadolinium oxide (Gd_2_O_3_); paramagnetic nanoparticles (PMNPs); iron oxide nanoparticles (IONPs); iodine nanoparticles (INPs); bismuth sulfide (Bi_2_S_3_); quantum dots (QDs); copper sulfide (CuS); polyethylene glycol (PEG); cyclic RGDfK peptide (cRGDfK); 1,2-Distearoyl-sn-glycero-3-phosphoethanolamine-N-amino(polyethylene glycol) (DSPE-PEG-NH2); superparamagnetic iron oxide (Fe_3_O_4_) nanoparticles (SPION) conjugated with plectin-1 antibody and Cy7 (Plectin-SPION-Cy7) (SPION-Cy7); Molybdenum trioxide (MoO_3_), Ruthenium(II)-bipyridine complex (Ru(II)), Ethylenediaminetetraacetic acid disodium salt (EDTA-2Na), Polypyrrole nanoparticles (PPy-NPs).
